# Over-the-counter Antifungal Therapy—A Word of Caution

**DOI:** 10.1155/S1064744995000123

**Published:** 1995

**Authors:** David E. Soper

**Affiliations:** Department of Obstetrics and Gynecology and Medicine Divsion of Infectious Diseases Medical College of Virginia Virginia Commonwealth University Richmond Virginia USA


Infectious Diseases in Obstetrics and Gynecology 2:249–250 (1995)
© 1995 Wiley-Liss, Inc.

### Over-the-Counter Antifungal Therapy—A Word of Caution

Major changes in the delivery of health care are occurring in an attempt to control runaway medical costs. One such change is the diversion of physician-prescribed therapeutics to over-the-counter (OTC) status, allowing the patient to self-diagnose and self-treat. The Federal Drug Administration (FDA) has adopted criteria, in addition to the usual evidence that the agent must be safe and effective, for OTC drugs to ensure the safety of their use. These criteria include consistency (a response highly likely to occur each time), simplicity (convenient for self-medication with one primary pharmacologic effect), and safety (no important interactions with other drugs, foods, or diseases). These standards and increasing postmarketing surveillance were designed to add important safety measures to prevent or control side effects associated with OTCs.[1]

Unfortunately, the standards neglect to address the accuracy of a patient's self-diagnosis. To what degree can we be guaranteed that patients with complaints of symptoms and self-detected signs of a lower genital tract infection will correctly diagnose themselves as having vulvovaginal candidiasis (VVC) as opposed to a potentially dangerous sexually transmitted disease (STD)? The symptoms of increased vaginal discharge and vulvar pruritus also characterize vaginitis secondary to *Trichomonas vaginalis*, a sexually transmitted parasite shown to be associated with *Neisseria gonorrhoeae* endocervical infection.[2] How often have clinicians heard their patients refer to their chief complaint of an abnormal vaginal discharge as "a yeast infection"? Would not a patient with an abnormal vaginal discharge due to mucopurulent endocervicitis secondary to *Chlamydia trachomatis* infection be falsely reassured if self-treatment with a vaginal medication temporarily improved her symptoms?

In the face of a continued epidemic of STDs, we would be more prudent to first appeal for more reliable diagnostics that could be used in the privacy of one's home to suggest the appropriate treatment of uncomplicated local infections. Moreover, prospective studies evaluating a patient's ability to accurately self-diagnose are in order. Most physicians have no concern with the low-risk patient, previously diagnosed in the office, utilizing an OTC antifungal when typical symptoms of a recurrent episode of VVC occur. However, the sexually active adolescent, fearful of an office visit and denying the possibility of exposure to an STD, may well utilize the same OTC products incorrectly. Let us ensure that we do not "put the cart before the horse," especially at a time when new pressure is being placed on the FDA to allow oral antibiotics to be available OTC.

*David E. Soper*
*Department of Obstetrics and Gynecology and Medicine*
*Division of Infectious Diseases*
*Medical College of Virginia*
*Virginia Commonwealth University*
*Richmond, Virginia*

Editorial

Received January 16, 1995
Accepted January 18, 1995

### REFERENCES

1. Wenzel RP, Kunin CM: Should oral antimicrobial drugs be available over the counter? J Infect Dis 170:1256–1259, 1994.
2. Wolner-Hanssen P, Krieger JN, Stevens CE, Kiviat NB, Koutsky L, Critchlow C, DeRouen T, Hillier S, Holmes KK: Clinical manifestations of vaginal trichomoniasis. JAMA 261:571–576, 1989.
